# Acute leukaemoid reaction following cardiac surgery

**DOI:** 10.1186/1749-8090-2-3

**Published:** 2007-01-09

**Authors:** Nigel E Drury, Ayyaz Ali, Shafi Mussa, Stephen T Webb, Kanchan P Rege, John Wallwork

**Affiliations:** 1 Department of Cardiac Surgery, Papworth Hospital, Cambridge, CB23 3RE, UK; 2 Department of Anaesthesia, Papworth Hospital, Cambridge, CB23 3RE, UK; 3 Department of Haematology, Papworth Hospital, Cambridge, CB23 3RE, UK

## Abstract

Chronic myelomonocytic leukaemia is an atypical myeloproliferative disorder with a natural history of progression to acute myeloid leukaemia, a complex and poorly understood response by the bone marrow to stress. Cardiac surgery activates many inflammatory cascades and may precipitate a systemic inflammatory response syndrome. We present a case of undiagnosed chronic myelomonocytic leukaemia who developed rapidly fatal multi-organ dysfunction following cardiac surgery due to an acute leukaemoid reaction.

## Background

Chronic myelomonocytic leukaemia (CMML) is an atypical myeloproliferative disorder characterized by an absolute peripheral blood monocyte count of greater than 1.0 × 10^9^/L, with evidence of both effective and ineffective haematopoiesis [1]. The aetiology of CMML is complex and incompletely understood. It is twice as common in men, usually presenting in the seventh or eighth decades. Late clinical features include fatigue, weight loss, fever and night sweats, splenomegaly and hepatomegaly, although it may present as a chance finding on blood analysis. The bone marrow is usually hypercellular with granulocytic proliferation and it can be differentiated from classical chronic myeloid leukaemia by the high monocyte count and absence of a Philadelphia chromosome. Despite best medical therapy, the outcome remains poor with median survival less than two years from the time of diagnosis. Individual prognosis is difficult to predict, although approximately 20% of patients progress to acute myeloid leukaemia [2].

Cardiac surgery has been reported in patients with chronic lymphocytic leukaemia, myelodysplasia and other malignant haematological disorders [3-5] but data on CMML are limited [6]. We present a case of previously undiagnosed CMML who developed fatal complications following cardiac surgery.

## Case report

A 70-year-old man was referred for coronary revascularization with angina (Canadian Cardiovascular Society class 2), three vessel disease and good left ventricular function. He was otherwise well, with no significant past medical history and physical examination was unremarkable. Preoperative risk assessment scores were: EuroSCORE standard 3 (age), logistic 1.82. Preoperative investigations revealed a white cell count (WCC) of 15.6 × 10^9^/L (neutrophils 10.1 × 10^9^/L, monocytes 2.5 × 10^9^/L) and urinalysis positive for blood. He denied any urinary or respiratory symptoms but was treated empirically for a lower urinary tract infection and discharged home. Urine culture was negative. Two weeks later, the WCC remained elevated at 14.2 × 10^9^/L (neutrophils 10.5 × 10^9^/L, monocytes 1.6 × 10^9^/L), although he remained asymptomatic and was keen to proceed. 

Coronary artery bypass grafting was performed using the left internal mammary artery and saphenous vein grafts.  Cardiopulmonary bypass (CPB) time was 76 minutes with aortic cross-clamp for 39 minutes, using antegrade cold crystalloid cardioplegia. The patient came off CPB easily and remained haemodynamically stable thereafter. He was extubated expeditiously and returned to the ward on the first postoperative day. Routine blood tests demonstrated a leukocytosis (46.9 × 10^9^/L) with marked neutrophilia (38.5 × 10^9^/L) and monocytosis (8.4 × 10^9^/L).

Late on the first postoperative day, his condition deteriorated with progressive hypoxia, oliguria and metabolic acidosis. He was readmitted to the intensive care unit for non-invasive ventilation and inotropic support. On the morning of the second postoperative day, he remained hypoxic and oliguric. A pulmonary artery catheter revealed low cardiac output state and low systemic vascular resistance. He was reintubated and mechanically ventilated, inotropic therapy was increased and continuous veno-venous haemofiltration and broad-spectrum antibiotics were commenced. A cardiac origin of low cardiac output was excluded and sepsis or splanchnic ischemia were considered as causes of his systemic inflammatory response syndrome (SIRS) and multi-organ dysfunction (MOD). Computed tomography of the thorax and abdomen showed bilateral ground glass shadowing of the lung parenchyma consistent with acute respiratory distress syndrome but no other significant findings. The leukocytosis increased to 91.7 × 10^9^/L (neutrophils 54.1 × 10^9^/L, monocytes 33.0 × 10^9^/L) and his condition deteriorated. A haematology opinion was sought and confirmed a diagnosis of probable leukaemoid reaction on a background of CMML. Despite maximal therapy, the patient died on the third postoperative day. Post-mortem findings were of an enlarged spleen and widespread SIRS, manifesting as pulmonary congestion with patchy hemorrhagic necrosis of the small and large bowel.

## Discussion

Cardiac surgery produces an altered activation of the inflammatory response due to the combination of surgical trauma, cardiopulmonary bypass and ischemia-reperfusion injury [7]. Marked amplification of this process can result in SIRS. In CMML, some patients may progress to the acute phase of myeloid leukaemia, a complex and poorly understood response by the bone marrow to stress. In this patient, it appears that the inflammatory storm generated by cardiac surgery with CPB precipitated a dramatic myelomonocytic leukaemoid reaction, with a clinical picture of SIRS and MOD. The pathways responsible for this response are unknown.

The patient was referred for cardiac surgery with undiagnosed CMML. As the proportion of elderly patients undergoing surgery steadily increases, more occult chronic conditions are likely to be diagnosed on preoperative work-up. Despite reports that patients with chronic haematological malignancies undergoing cardiac surgery are at increased risk of complications, including bleeding and infection [3], a preoperative diagnosis would not necessarily have altered the decision to proceed with surgical revascularization. In retrospect, the blood count and film were suggestive of CMML in the chronic phase, with no evidence of acute transformation or the leukaemoid reaction to come. Indeed, urgent coronary artery surgery has been successfully performed in a patient with acute myeloid leukaemia [8]. The key questions are whether patients with haematological malignancies with a poor prognosis should be candidates for cardiac surgery for prognostic benefit *per se *and why our patient responded to the inflammatory stress of surgery in such a catastrophic and unpredictable fashion. This may be related to the unfortunate timing of surgery in the natural course of his disease or the susceptibility of his particular subtype of CMML to inflammatory stress.

Patients with CMML may benefit from a strategy aimed at decreasing their exposure to inflammatory stimuli. Off-pump coronary artery bypass (OPCAB) surgery avoids the need for CPB but still causes significant surgical trauma. Whilst recent studies suggest a decrease in the systemic inflammatory response in OPCAB surgery, there may not always be a direct correlation between measured inflammatory markers, pathophysiological consequences and clinical outcome [7]. The only report of OPCAB in a patient with CMML, performed via a median sternotomy, highlighted concerns over leukaemoid transformation and the assumed benefits of a reduction in cytokine release [6].

In conclusion, patients with CMML are at increased risk for cardiac surgery. The response to the inflammatory stimulus of surgery is difficult to predict. A leukaemoid reaction may prove rapidly fatal although the cause of disease acceleration and potential remitting factors remain obscure. With better understanding of the inflammatory response to surgery, cardiopulmonary bypass and bone marrow homeostasis in chronic leukaemic conditions, patient selection for surgery may be refined dependent on CMML subtype risk assessment. As these patients may represent a group in whom the lowering of inflammatory stress is particularly beneficial, OPCAB may have a specific role. Alternatively, cytokines such as interleukin-2 and interleukin-6 may present potential therapeutic targets for modulating the inflammatory response following cardiac surgery in patients at risk.

## Competing interests

The author(s) declare that they have no competing interests.
